# Extracellular matrix stiffness dictates Wnt expression through integrin pathway

**DOI:** 10.1038/srep20395

**Published:** 2016-02-08

**Authors:** Jing Du, Yan Zu, Jing Li, Shuyuan Du, Yipu Xu, Lang Zhang, Li Jiang, Zhao Wang, Shu Chien, Chun Yang

**Affiliations:** 1 Institute of Biomechanics and Medical Engineering, School of Aerospace, Tsinghua University, Beijing 100084, P.R. China; 2 Departments of Bioengineering and Medicine, and Institute of Engineering in Medicine, University of California at San Diego, La Jolla, CA 92093; 3 School of Stomatology, Lanzhou University, 730000, P.R. China; 4 Department of Pharmacology, School of Medicine, Tsinghua University, Beijing 100084, P.R. China

## Abstract

It is well established that extracellular matrix (ECM) stiffness plays a significant role in regulating the phenotypes and behaviors of many cell types. However, the mechanism underlying the sensing of mechanical cues and subsequent elasticity-triggered pathways remains largely unknown. We observed that stiff ECM significantly enhanced the expression level of several members of the Wnt/β-catenin pathway in both bone marrow mesenchymal stem cells and primary chondrocytes. The activation of β-catenin by stiff ECM is not dependent on Wnt signals but is elevated by the activation of integrin/ focal adhesion kinase (FAK) pathway. The accumulated β-catenin then bound to the wnt1 promoter region to up-regulate the gene transcription, thus constituting a positive feedback of the Wnt/β-catenin pathway. With the amplifying effect of positive feedback, this integrin-activated β-catenin/Wnt pathway plays significant roles in mediating the enhancement of Wnt signal on stiff ECM and contributes to the regulation of mesenchymal stem cell differentiation and primary chondrocyte phenotype maintenance. The present integrin-regulated Wnt1 expression and signaling contributes to the understanding of the molecular mechanisms underlying the regulation of cell behaviors by ECM elasticity.

It has become increasingly apparent that each tissue has a characteristic ‘stiffness phenotype’. All cells in tissues and organs are exposed to ECM stiffness and specifically tuned to the stiffness of the particular tissue in which it resides^1^,^2^. ECM stiffness, being a mechanical property, exerts its effects on a variety of cell behaviors such as proliferation, differentiation, apoptosis, organization, and migration^3^,^4^,^5^,^6^,^7^,^8^. The mechanical cues of ECM stiffness sensed by the cell are propagated, amplified, and transduced into signaling cascades to lead to transient or sustained cellular responses. Previous reports have demonstrated that ECM stiffness regulates cells function via its impact on the contraction force in actomyosin fibers, the subcellular allocation of integrin, and the PI3K pathways^9^,^10^,^11^. Despite these findings, how ECM stiffness forges significant effect on different types of cells remains unsolved. In particular, the mechanisms of stiffness sensing and the downstream signal transduction involved in the ensuing gene regulation are yet to be clarified.

Recent reports have shown that Wnt signaling is responsive to matrix rigidity^12^. Our microarray screening results revealed a significant promotion of canonical Wnt/β-catenin pathway by the stiffer ECM, which was confirmed by Western blotting. The Wnt/β- catenin pathway is a rather ubiquitous mechanism in controlling diverse cell functions and behaviors including cell adhesion, migration, differentiation, and proliferation, and these cellular behaviors respond significantly to ECM stiffness^13^. We thus aimed to explore the mechanism of regulation of Wnt expression and its role in cellular stiffness sensing. Our results showed that the promotion of canonical Wnt/β-catenin pathway by stiff ECM was not dependent on Wnt *per se*, but caused by the accumulation of β-catenin induced by the activation of integrin/ focal adhesion kinase (FAK) pathway. β-catenin in turn activated the expression of Wnt1 by binding to the promoter region of *wnt1* gene and promotes the gene transcription. The integrin-activated β-catenin/Wnt pathway connects with canonical Wnt/β-catenin pathway to form a positive feedback loop, which is crucial to the promotion of Wnt signal by stiff ECM and the regulation of mesenchymal stem cells differentiation and primary chondrocytes phenotype maintenance.

## Results

### Effects of ECM Stiffness on Wnt and β-catenin

Cultured chondrocytes for implantation or engineered cartilage on scaffolds are potential therapies to the articular cartilage repair. Previous findings indicated that chondrocytes cultured *in vitro* are sensitive to the elasticity of the substrate coated by type I collagen (ColI), a widely used ECM mimic in the study of the effect of substrate stiffness on many cell types^14^,^15^. This suggests that the substrate elasticity is crucial in engineered cartilage, and arose a question on the underlying mechanism of chondrocytes sensing elasticity.

*In vivo* chondrocytes were embedded in pericellular matrix (PCM) of which the mechanical property is crucial in the environment of the chondrocyte^16^. The Young’s modulus of the enzyme-isolated PCM (1–2 kPa) was 1–2 orders of magnitude lower than that of the cartilage ECM^17^. Thus, we cultured the chondrocytes on ColI-coated soft (0.5–1 kPa) and/or stiff (100 kPa) substrate to explore the elastic sensing pathway of chondrocytes.

**Figure 1:**
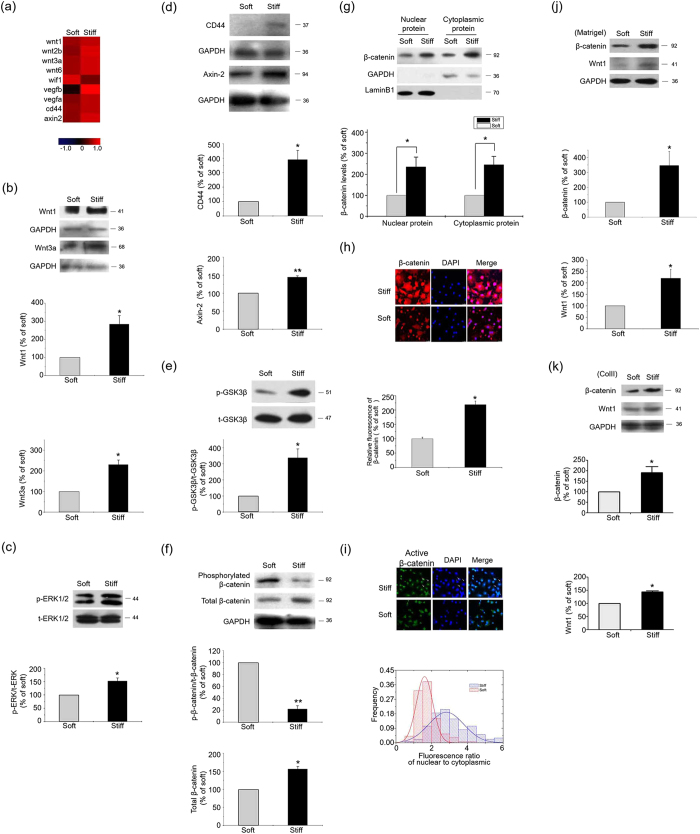
Wnt/β-catenin pathway was activated by the stiff ECM. Results from primary chondrocytes 48 hr after seeding on stiff (100 kPa) or soft (0.5–1 kPa) ECM. (**a**) Microarray profiling of Wnt/β-catenin pathway transcripts. Results are normalized by median scaling using Rosetta Resolver System software. (**b**) Wnt1 and Wnt3a levels were analyzed by western blotting. (**c**) Total and phosphorylated ERK1/2 levels were analyzed by western blotting. (**d**) Axin2, CD44, and (**e**) phosphorylated GSK3β levels were analyzed by western blotting. (**f**) Total and phosphorylated β−catenin levels were analyzed by western blotting. (**g**) β−catenin levels in nucleus and cytoplasm were analyzed by western blotting. (**h**) Total and (**i**) activated β-catenin levels and distribution in chondrocytes 2 hr after seeding on stiff or soft ECM were analyzed by *in situ* fluorescence staining. (**j**) β-catenin and wnt1 levels in chondrocytes 48 hr after seeding on the Matrigel-coated PAAM were analyzed by western blotting. (**k**) β-catenin and wnt1 levels in chondrocytes 48 hr after seeding on the ColII-coated PAAM were analyzed by western blotting. Western results were from 3 independent experiments for each individual protein, with blots exemplifying one experiment and the bar graphs showing the combined results of 3 experiments on stiff matrix expressed as percentages (mean ± SEM) of the corresponding results on the soft matrix. GAPDH was used to normalize for equal loading. *P < 0.05, **P < 0.01. n.s. stands for not statistically significant.

Microarray analyses demonstrated that genes were significantly regulated by the substrate stiffness (Supplementary table 1). Among these genes, several members of Wnt family, such as *wnt1, wnt3a* and canonical Wnt/β-catenin pathway target genes, were up-regulated by the stiff ECM in comparison with the soft ECM, at both mRNA (Fig. 1a) and protein levels (Fig. 1b–d). By binding to the cognate Frizzled receptors, Wnt proteins transduce their signals through dishevelled proteins to inhibit glycogen synthase kinase 3β (GSK3β), leading to the accumulation of cytosolic β-catenin^13^,^18^,^19^. The stiff ECM led to an increase in GSK3β phosphorylation on Ser9 (Fig. 1e), a reduction of β-catenin phosphorylation, and a rise in both total β-catenin and nuclear β-catenin (Fig. 1f,g). An *in situ* fluorescence staining of total and activated β-catenin showed that the stiff ECM led to a high fluorescence intensity of total β-catenin and an increase of activated β-catenin in nucleus when compared to the soft ECM (Fig. 1h,i). These findings suggest that stiff ECM activates the Wnt and β-catenin pathways.

To test whether the effect of ECM stiffness on Wnt/β-catenin pathway is a unique phenomenon caused by ColI or a general mechanism, we employed other substrate proteins, including type II collagen (ColII) and matrigel, two frequently used ECM mimics in engineered cartilage, to modify the PAAM gel. The stiffness of the ColII or matrigel-coated substrates also up-regulated the levels of Wnt1 and β-catenin in chondrocytes, as ColI (Fig. 1j,k). These results suggest that the effect of substrate stiffness on chondrocytes is demonstrable with many frequently used ECM factors. In the following study, the ColI-coated PAAM system was used for its wide usage in exploring the mechanism by which cells sense and tune to ECM stiffness.

### The Effect of ECM Stiffness on β-catenin is in Upstream of Wnt Signals

**Figure 2:**
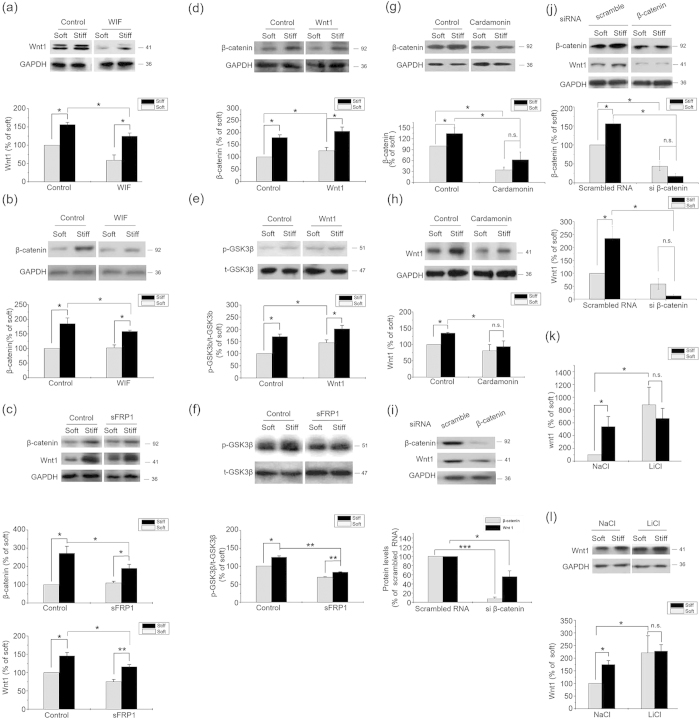
ECM stiffness regulated β-catenin accumulation independently with Wnt signals. Results from primary chondrocytes 48 hr after seeding on stiff (100 kPa) or soft (0.5–1 kPa) ECM. (**a**) Wnt1 and (**b**) β-catenin levels in the presence of WIF-1 (1 μg/ml) or solvent were analyzed by western blotting. (**c**) Total β-catenin and wnt1 levels in the presence of sFRP1 (1 μg/ml) or solvent were analyzed by western blotting. (**d**) Total β-catenin and (**e**) phosphorylated GSK3β levels in the presence of Wnt1 (100 ng/ml) or solvent were analyzed by western blotting. (**f**) Phosphorylated GSK3β levels in the presence of sFRP1 (1 μg/ml) or solvent were analyzed by western blotting. (**g**) β-catenin and (**h**) Wnt1 levels in chondrocytes treated with 10 μM Cardamonin or DMSO were analyzed by western blotting. (**i**) β-catenin and Wnt1 levels in chondrocytes transfected by β−catenin siRNA or scrambled siRNA on normal plates and (**j**) on stiff or soft ECM. (**k**) Wnt1 mRNA levels in chondrocytes treated with NaCl or LiCl (20 mmol/L) were analyzed by Real-time PCR. (**l**)Wnt1 levels in chondrocytes treated with NaCl or LiCl (20 mmol/L) were analyzed by western blotting. Western results were from 3 independent experiments (except 5 in b and i, 6 in j), with blots exemplifying one experiment and the bar graphs showing the combined results of 5 experiments as percentages (mean ± SEM) of the corresponding results on soft matrix. GAPDH was used to normalize for equal loading in western blotting. *P < 0.05, **P < 0.01, n.s. stands for not statistically significant.

To evaluate the effect of Wnt signals on cells cultured on substrate with different stiffness, we assayed the β-catenin protein level of the cells treated with WIF-1, sFRP1, or Wnt1. Neither inhibition (with WIF-1 and sFRP1)^20^ nor induction (with Wnt1) of Wnt signals significantly altered the differences in the levels of Wnt1, β-catenin, and phosphorylated GSK3β between the stiff and the soft ECMs (Fig. 2a–f), suggesting that ECM stiffness-induced β-catenin accumulation is Wnt independent. However, the increase of Wnt1 expression on the stiff ECM was blocked by cardamonin^21^,^22^, a Wnt-independent inhibitor of β-catenin (Fig. 2g,h). A more specific inhibition by β-catenin siRNA significantly down-regulated the Wnt1 expression and diminished the difference of Wnt1 levels in cells on soft and stiff substrate. (Fig. 2i,j). LiCl, a β-catenin activator, significantly induced Wnt1 mRNA (Fig. 2k) and protein expressions (Fig. 2l) in cells cultured on the soft ECM, and diminished the difference in Wnt1 expression between stiff and soft ECMs (Fig. 2l). These results suggest that the increase of Wnt1 expression on stiff substrate is mediated by β-catenin, which may be the upstream of Wnt1.

### Binding of β-catenin to Wnt1 Promoter Region as a Novel Transcriptional Mechanism of Wnt1

**Figure 3:**
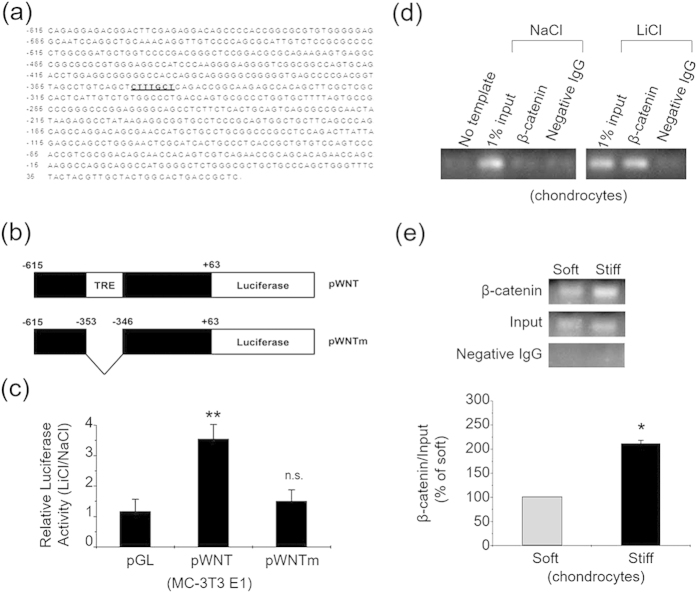
A novel feedback control mechanism of Wnt/β-catenin pathway. (**a**) Upstream sequence of mouse *wnt1* gene. Putative TRE is shaded. (**b**) Construct map of the luciferase reporter constructs used in luciferase activity assays. Constructs contain various portions of mouse *wnt1* gene upstream sequence, as indicated. The putative TRE is denoted by a shaded box. (**c**) MC-3T3 cells were transfected with the reporter constructs pWNT, pWNTm and the control construct pGL_3_-Basic (pGL). The cells were then treated with either 20 mM LiCl or NaCl. Bar graph shows the results (mean ± SEM) on luciferase activities for transfection with control plasmid (pGL), pWNT or pWNTm, with 3 experiments in each group. (**d**) Primary chondrocytes were treated with 20 mM LiCl or NaCl for 24 hr, and then ChIP assays were performed. No template: PCR amplification without **DNA** sample; 1% input: samples representing total input chromatin (1%) for each experiment; negative IgG: immunoprecipitated with negative IgG instead of β-catenin antibody. (**e**) Primary chondrocytes cultured on the stiff or soft ECM for 24 hr were subjected to quantitative ChIP assays. ChIP results were from 3 independent experiments, and the bar graphs showing the combined results of 3 experiments on the stiff matrix as percentages (mean ± SEM) of the corresponding results on the soft matrix. *P < 0.05, **P < 0.01, n.s. stands for not statistically significant.

Bioinformatics study predicted a β-catenin /TCF-responsive element (TRE) in the mouse *wnt1* gene promoter region (Fig. 3a). LiCl treatment significantly induced the transcriptional activity of the putative TRE-containing luciferase reporter (pWNT) in MC3T3, but not the TRE-deleted mutant construct (pWNTm) (Fig. 3b,c). Chromatin immunoprecipitation (ChIP) results indicated that upon activation by LiCl, β-catenin in primary chondrocytes is bound to the *wnt1* promoter sequence flanking the putative TRE (Fig. 3d). Quantitative ChIP assay also indicated a significant enhancement of the binding of β-catenin to the *wnt1* promoter region on the stiff ECM (Fig. 3e). These results, together with the β-catenin and Wnt inhibition results, provide a novel transcriptional mechanism of Wnt1 protein.

### Role of Integrin in the Activation of β-catenin and Wnt on Stiff ECM

We then proceeded to investigate the molecular mechanism by which stiff ECM triggers the Wnt-independent activation of β-catenin.

**Figure 4:**
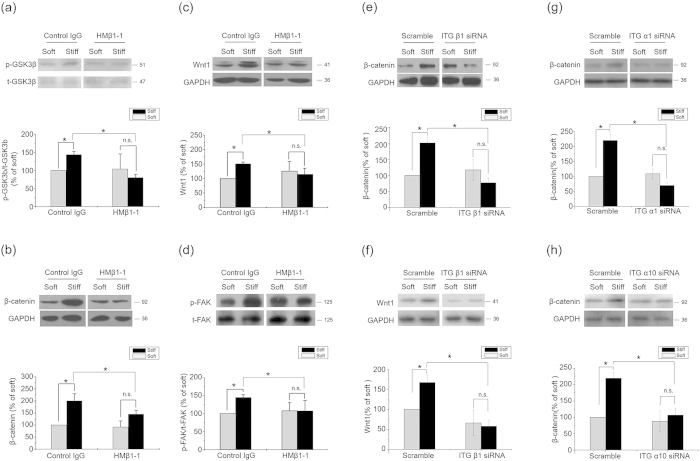
ECM regulated β-catenin pathway and Wnt expression by Integrins. Results from primary chondrocytes 48 hr after seeding on stiff (100 kPa) or soft (0.5–1 kPa) ECM. (**a**) Phosphorylated GSK3β levels, (**b**) β-catenin and (**c**) Wnt1 levels in the presence of 20 μg/ml β1 integrin blocking antibody or control antibody were analyzed by western blotting. (**d**) Phosphorylated FAK levels in the presence of 20 μg/ml β1 integrin blocking antibody or control antibody were analyzed by western blotting. (**e**) β-catenin and (**f**) Wnt1 levels in the presence of integrin β1 siRNA or scramble were analyzed by western blotting. (**g**) β-catenin in the presence of integrin α1 siRNA or scramble were analyzed by western blotting. (**h**) β-catenin levels in chondrocytes 48 hr after seeding on stiff or soft ECM in the presence of integrin α10 siRNA or scramble were analyzed by western blotting. Western results were from 3 independent experiments (except 4 in b, 6 in c), with blots exemplifying one experiment and the bar graphs showing the combined results on stiff matrix expressed as percentages (mean ± SEM) of the corresponding results on soft matrix. GAPDH was used to normalize for equal loading in western blotting. *P < 0.05, **P < 0.01, n.s. stands for not statistically significant.

In light of the promoting effects of stiff ECM on cell membrane integrin activity^23^ and the regulation of β-catenin accumulation by integrin signals^24^, we investigated the involvement of integrin and its downstream signals in the activation of β-catenin/Wnt pathway on the stiff ECM. Considering that α1β1 and α10β1 were collagen-binding integrins, we used α1β1 and α10β1 siRNA, respectively, to study the role of integrins in the regulation of Wnt signaling^25^. The inhibition of β1 integrin by a functional blocking antibody (clone HMβ1-1) significantly diminished the influence of ECM stiffness on the levels of phosphorylated GSK3β, β-catenin and Wnt1 (Fig. 4a–c). A more specific knockdown of β1 integrin by siRNA not only down regulated but also wiped off the effect of ECM stiffness on the levels of β-catenin and Wnt1 (Fig. 4e,f). The interference of α1 and α10 integrins also down regulated the levels of β-catenin (Fig. 4g,h). These results suggested a crucial role of integrins in activating the β-catenin pathway and the ensuing Wnt expression on stiff ECM.

**Figure 5:**
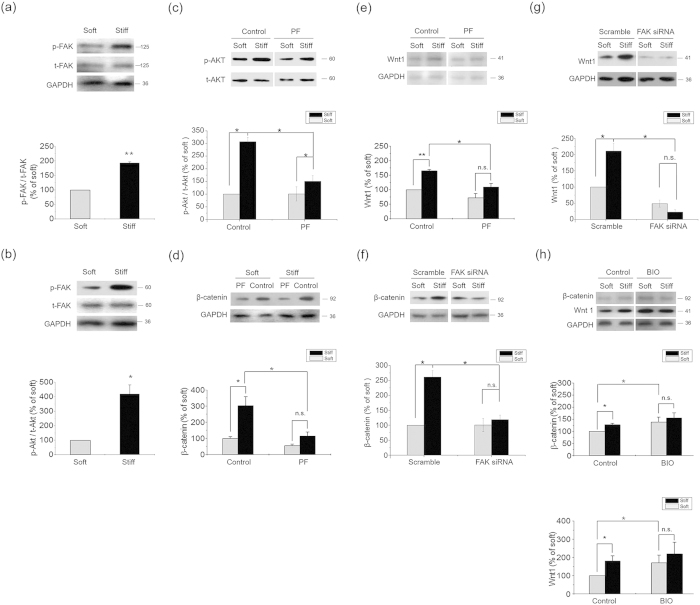
ECM stiffness regulated β-catenin accumulation via integrin/FAK/Akt pathway. Results from primary chondrocytes 48 hr after seeding on stiff (100 kPa) or soft (0.5–1 kPa) ECM. (**a**) Phosphorylated FAK and (**b**) Phosphorylated Akt levels were analyzed by western blotting. (**c**) Phosphorylated Akt levels in the presence of 1 μM PF573228 or DMSO were analyzed by western blotting. (**d**) β-catenin and (**e**) Wnt1 levels in the presence of 1 μM PF573228 or DMSO were analyzed by western blotting. (**f**) β-catenin and (**g**) Wnt1 levels in the presence of FAK siRNA or scramble were analyzed by western blotting. (**h**) Total β-catenin and Wnt1 levels in the presence of BIO (10 μM) or DMSO were analyzed by western blotting. Western results were from 3 independent experiments (except 6 in f–h), with blots exemplifying one experiment and the bar graphs showing the combined results on stiff matrix expressed as percentages (means ± SEM) of the corresponding results on soft matrix. GAPDH was used to normalize for equal loading in western blotting. *P < 0.05, **P < 0.01, n.s. stands for not statistically significant.

As an important downstream element of integrin signals, FAK/Akt pathway is well documented as a regulator of GSK3β. Τhe stiff ECM significantly induced the phosphorylation levels of both FAK (Fig. 5a) and Akt (Fig. 5b). The inhibition of β1 integrin significantly diminished the influence of ECM stiffness on the level of FAK phosphorylation (Fig. 4d). Inhibition of FAK by a specific inhibitor PF573228 also significantly reduced Akt phosphorylation (Fig. 5c), β-catenin accumulation (Fig. 5d) and Wnt1 expression (Fig. 5e). Knocking down FAK by siRNA wiped off the effects of ECM stiffness on β-catenin accumulation (Fig. 5f) and Wnt1 expression (Fig. 5g). Inhibition of Akt activity by its inhibitor Akti-1/2 reduced, though did not totally block the differences in the levels of β-catenin and Wnt1 between the stiff and the soft ECMs (supplementary Fig. 1a,b). In addition, integrin linked kinase (ILK), PKC, as well as YAP/TAZ represent additional targets for integrin signaling^26^,^27^. These proteins also regulate GSK3β phosphorylation, and influence the subsequent β-catenin signaling pathways^28^. We thus assayed β-catenin level and GSK-3β phosphorylation of the cells treated with PMA (the activator of PKC), and the siRNAs of ILK and YAP /TAZ. We found that PMA up-regulated the accumulation of β-catenin and phosphorylate GSK-3β on stiff substrate, but had no significant influence on soft substrate (supplementary Fig. 1c). ILK siRNA treatment down-regulated β-catenin levels and GSK-3β phosphorylation on both stiff and soft substrates, but failed to eliminate the stiffness-induced differences of these two proteins (supplementary Fig. 1d). The interference of YAP/TAZ also down-regulated β-catenin levels and GSK-3β phosphorylation on both stiff and soft substrates, and diminished the stiffness-induced differences of GSK-3β phosphorylation (supplementary Fig. 1e). However, knocking down YAP/TAZ could not eliminate the difference of β-catenin accumulation induced by substrate stiffness (supplementary Fig. 1e). These results suggest that in chondrocytes, PKC, ILK, YAP/TAZ have no significant contribution to the difference of β-catenin signaling induced by substrate stiffness.

Finally, BIO, an inhibitor of GSK3β, significantly diminished the influence of ECM stiffness on Wnt1 expression, as well as β-catenin accumulation (Fig. 5h). These results indicate that the β-catenin/Wnt pathway is activated by stiff ECM via inhibition of GSK3β by integrin-dependent downstream signals.

### Effect of the integrin-activated β-catenin/Wnt pathway on the functional responses of primary chondrocytes and BMMSCs to ECM stiffness

**Figure 6:**
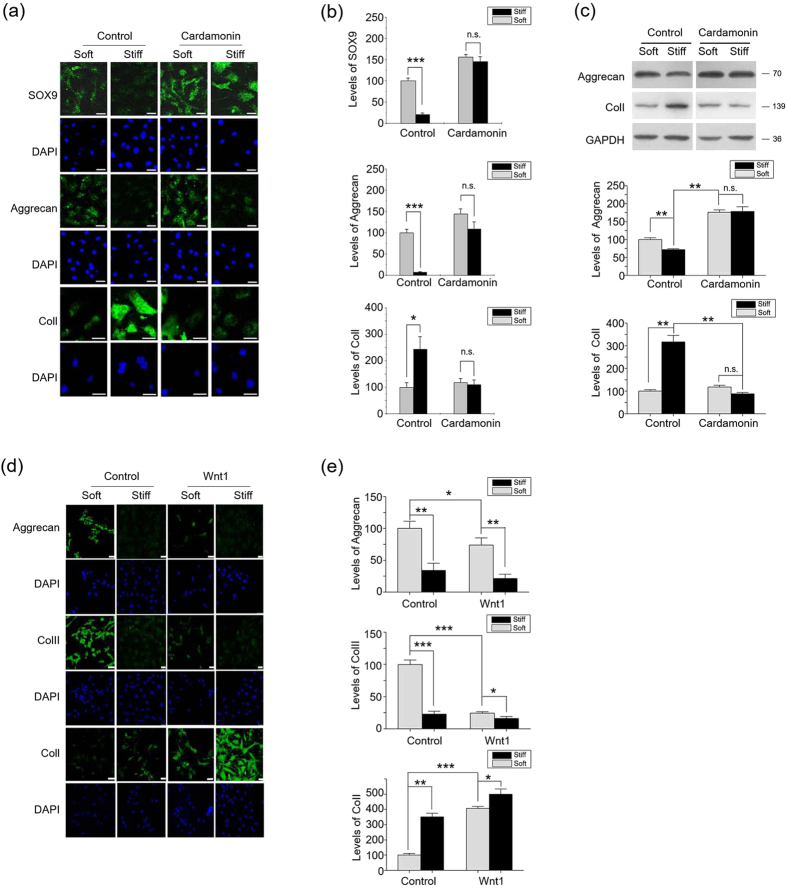
Wnt/β-catenin pathway contributes to ECM stiffness regulating chondrocytes phenotype maintenance. (**a**) Chondrocytes were cultured on stiff or soft ECM for seven days in the presence of 10 μM Cardamonin or DMSO, followed by determination of Sox9, Aggrecan and ColI expressions by immunocytochemical staining. Scale Bar: 30 μm. (**b**) represents the statistical results of (**a**). (**c**) Chondrocytes were cultured on stiff or soft ECM for seven days in the presence of 10 μM Cardamonin or DMSO, followed by determination of Aggrecan, ColII and ColI expressions by western blotting. (**d**) Chondrocytes were cultured on stiff or soft ECM for seven days in the presence of Wnt1 (100 ng/ml) or solvent, followed by determination of Aggrecan, ColII and ColI expressions by immunocytochemical staining. Scale Bar: 30 μm. (**e**) represents the statistical results of (**d**). Scale Bar: 30 μm. Results on fluorescence intensities from 6 independent experiments on stiff matrix are expressed as percentages (mean ± SEM) of the corresponding results on the soft matrix. Western results were from 3 independent experiments, with blots exemplifying one experiment and the bar graphs showing the combined results on stiff matrix expressed as percentages (means ± SEM) of the corresponding results on soft matrix. GAPDH was used to normalize for equal loading in western blotting. *P < 0.05, **P < 0.01, ***P < 0.001, n.s. stands for not statistically significant.

We then explored the effect of the integrin-activated β-catenin/Wnt pathway on the functional responses of primary chondrocytes and bone marrow mesenchymal stem cells (BMMSCs) to ECM stiffness. Chondrocytes, which are surrounded by soft (1–2 kPa) pericelluar matrix ECM, demonstrate compliance-dependent behavior^15^,^29^. Stiff ECM is known to induce a significant de-differentiation of monolayer cultured chondrocytes with decreases of Sox9 and Aggrecan and an increase of ColI expression. β-catenin inhibitor cardamonin blocked the de-differentiation of chondrocytes on the stiff ECM (Fig. 6a–c), while Wnt1 and Wnt3a inhibited the phenotype maintenance on the soft ECM through down-regulating Aggrecan and ColII expressions (Fig. 6d,e, supplementary Figure. 2a–c). These data suggest an important role of the β-catenin/Wnt pathway in the phenotype maintenance of chondrocytes by ECM stiffness.

**Figure 7:**
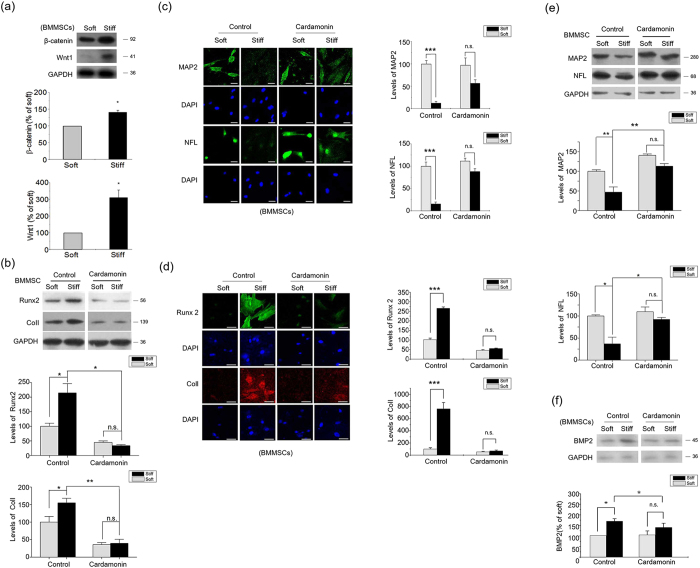
Wnt/β-catenin pathway contributes to the regulation of BMMSC Differentiation by ECM stiffness. (**a**) β-catenin and Wnt1 levels in BMMSCs 48 hr after seeding on stiff or soft ECM were analyzed by western blotting. (**b**) BMMSCs were cultured on stiff or soft ECM for seven days in the presence of 10 μM Cardamonin or DMSO, followed by determination of Runx2, ColI expressions western blotting. (**c,d**) BMMSCs were cultured on stiff or soft ECM for seven days in the presence of 10 μM Cardamonin or DMSO, followed by determination of MAP2, NFL, Runx2 and ColI expressions by immunocytochemical staining. Scale Bar: 30 μm. (**e**) BMMSCs were cultured on stiff or soft ECM for seven days in the presence of 10 μM Cardamonin or DMSO, followed by determination of MAP2 and NFL expressions western blotting. (**f**) BMP2 levels in BMMSCs treated with 10 μM Cardamonin or DMSO for 24 hr were analyzed by western blotting. Results on fluorescence intensities from 6 independent experiments on stiff matrix are expressed as percentages (mean ± SEM) of the corresponding results on the soft matrix. Western results were from 3 independent experiments with blots exemplifying one experiment and the bar graphs showing the combined results of 3 experiments on stiff matrix expressed as percentages of the corresponding results on the soft matrix. GAPDH was used to normalize for equal loading. *P < 0.05, **P < 0.01, ***P < 0.001, n.s. stands for not statistically significant.

It is well recognized that changing ECM stiffness has profound impact on the linage specification of stem cells^30^. A stiff ECM microenvironment drives BMMSCs to undergo osteogenic lineage specification, while a soft ECM favors neural lineage commitment^7^. In BMMSCs, the levels of β-catenin and Wnt1 were promoted by the stiff ECM (Fig. 7a). Furthermore, the inhibition of β-catenin significantly repressed osteogenic differential markers (Runx2 and ColI) (Fig. 7b,d), while inducing neural linage markers (MAP2 and NFL) on the stiff ECM (Fig. 7c,e). These results indicate that the β-catenin/Wnt pathway triggered by ECM stiffness has a crucial effect on the mechanical sensing by BMMSCs.

The present work demonstrates that the integrin/FAK-activated β-catenin/Wnt pathway plays crucial roles in the mechanical sensing processes of chondrocytes and stem cells. We have previously shown that ECM stiffness modulates stem cell linage commitment through a BMP-dependent pathway, which is known to crosstalk with Wnt signals^31^, and BMP2 is reported to be transcriptionally activated by β-catenin. In the present work, we also found an up-regulation of BMP2 (Fig. 7f) in BMMSCs on the stiff ECM, suggesting that crosstalking between BMP and Wnt pathways may contribute to the linage commitment of stem cells on ECMs of different stiffness.

## Discussion

Wnt proteins are a large family of 19 secreted glycoproteins that trigger multiple signaling cascades essential for embryonic development and tissue regeneration^32^. Microarray analyses demonstrated that *wnt1, wnt2b, wnt3a, wnt6* were significantly regulated by the substrate stiffness. Previous study demonstrated that Wnt1, Wnt2b and Wnt3a can regulate chondrocyte differentiation and maturation through the canonical β-catenin pathway, while Wnt6 mediated chondrocyte differentiation through Wnt/Ca^2+^ signaling^33^. Being rather ubiquitous in different cell types, the canonical Wnt/β-catenin pathway plays an important role in cell adhesion, migration, differentiation, and proliferation^13^,^34^,^35^. Here, we showed that the Wnt/β-catenin pathway regulates the responses of cell functions to ECM stiffness, at least in two cell types (chondrocytes and mesenchymal stem cells).

There is substantial knowledge on the signaling cascade downstream to Wnt stimulation; however, the control of production of Wnt proteins has remained an essentially uncharted territory^36^,^37^. By investigating the mechanotransduction pathways of cells sensing ECM elasticity, we found that both the pharmacological and genetic perturbation to β-catenin significantly down regulated the Wnt1 expression. Luciferase activity assays and chromatin immunoprecipitation results indicated a novel *wnt1* transcriptional mechanism, in which β-catenin regulates *wnt1* expression via binding to the promoter region. These results suggest that the canonical Wnt/β-catenin pathway contribute to stiff ECM inducing β-catenin accumulation, thus synergizing with the β-catenin-induced transcription of *wnt1* and form a positive feedback loop.

β-catenin/Wnt has been found to be an auto-regulated pathway at many other levels. The expressions of Wnt canonical receptors, LRP and Frizzleds have been reported to be controlled by β-catenin/TCF complex and contribute to the positive feedback loop of β-catenin/Wnt pathway^34^. There also exist negative feedback loops, which are essential in the maintenance of the stable levels of Wnt signals. Axin2 and Dkk4 have been identified as Wnt target genes, and they constitute negative feedback loops via negatively regulating Wnt pathway^13^,^35^. By demonstrating a new positive feedback loop, the present work contributes to the knowledge of Wnt regulating loops.

The integrin family is composed of 24 αβ heterodimeric members that involved in processes of cell-cell and cell-extracellular matrix adhesion and signal transduction. Integrin triggers a cascade of signaling pathways, including FAK, integrin-linked kinase, PKC, and YAP/TAZ phosphorylation, which are closely associated with osteogenic cell differentiation, bone formation and repair. The key integrin ligands in osteoblastic or osteocytic cells include αvβ1, αvβ3, α2β1, α4β1, α5β1. Articular chondrocytes were found to predominately express α1β1, α3β1, α5β1, and α10β1 integrins both *in vitro* and *in situ*. Considering that α1β1 and α10β1 were collagen-binding integrins, we used α1β1 and α10β1 siRNA, respectively, to study the role of integrins in the regulation of wnt signaling. Our results showed that integrin is activated on stiff ECM to phosphorylate downstream elements, leading to GSK3β-phosphorylation. As a result, β-catenin is prevented from degradation and translocates into the nucleus to bind with the *wnt1*-promoter to start the β-catenin/Wnt1 feedback. By enhancing or amplifying Wnt signals, the positive feedback loop initiated by integrin/FAK in response to stiff ECM may provide a key process for the activation of the β-catenin/Wnt pathway.

The downstream regulatory pathways of integrin may branch off at many steps. For instance, we observed that the inhibition of Akt only partially block the effect of ECM stiffness on β-catenin pathway. The up-regulation of ERK on stiff ECM and the co-inhibiting effect of ERK and Akt suggest co-regulatory effects of them on the integrin-initiated β-catenin/Wnt feedback on stiff ECM. However, the blocking experiments on integrin-linked kinase, PKC, and YAP/TAZ showed that these elements have no significant impact on the stiffness-induced difference of β-catenin signaling.

Taken together, mechanical stimuli and Wnt signals play significant roles in development, adult tissue homeostasis, and diseases^4^,^13^,^34^,^38^. The present pathway helps to shed light on understanding the regulatory effect of mechanical stimuli in these processes, and may provide therapeutic targets for related diseases and tissue engineering.

## Methods

### Cell Culture

Primary mouse knee chondrocytes were isolated and cultured from one-day-old Kunming mice. In brief, the articular cartilage was isolated and chondrocytes were extracted by collagenase digestion. After configuring the chondrocytes were plated in monolayer culture in DMEM high glucose supplemented with 10% FBS, 1% glutamine, 1% penicillin-streptomycin at 37 °C in 5% CO_2_. Primary BMMSC culturing was performed according to Tang *et al.*^39^, with slight modifications. One-month-old Sprague Dawley rats were sacrificed and sterilized by immersion in 75% ethanol/water for 5–10 min. Bone marrow was obtained from femurs and tibias. BMMSCs were extruded, isolated by Percoll density gradient centrifugation (1.073 g/L), and cultured in DMEM-F12 medium with the addition of 10% FBS, 2 mmol/L L-glutamine and 1% penicillin-streptomycin at 37 °C and 5% CO_2_.

MC3T3-E1 osteoblastic cells were bought from the Cell Center in School of Basic Medicine of Peking Union Medical College and cultured in DMEM medium with the addition of 10% FBS, 2 mmol/L L-glutamine and 1% penicillin-streptomycin, at 37 °C and 5% CO_2_.

All animal experiments were approved by the guidelines of the Animal Care and Experimentation Committee of Tsinghua University, China and in accordance with the approved guidelines of Tsinghua University.

### Substrate Preparation

Polyacrylamide gels with variable Young’s moduli were prepared according to a previously established protocol by Engler *et al.*^40^. Briefly, acrylamide and bis-acrylamide mixture with indicated concentrations was allowed to polymerize on a glass slide, and the gel was then covered by sulfosuccinimidyl-6-[4′-azido-2′-nitrophenylamino] hexanoate (Sulfo-SANPAH; Pierce). After exposure to UV light for 10 min twice, the polyacrylamide sheet was washed twice and incubated with a solution of type I collagen (0.2 mg/ml; Gibco BRL, Gaithersburg, MD), type II collagen (0.01 mg/ml; Sigma-Aldrich, St. Louis, MO, USA), or matrigel (1:100; BD Biosciences, San Jose, CA, USA) overnight at 4 °C. The elasticity moduli of the soft and the stiff gels were 500 Pa and 10^5^ Pa, respectively.

### Microarray analyses

Microarray analyses were performed using commercial Mouse cDNA Microarray slides (Phalanx Biotech Group; Hsinchu, Taiwan) according to the manufacturer’s instructions. Briefly, cells were collected and total mRNA was isolated with Trizol (Invitrogen, Carlsbad, CA, USA). The concentration, purity, and integrity (RIN) of the RNA extract were determined by spectrophotometry (Nanodrop 1000) and the Agilent RNA 6000 Nano assay, respectively. aaRNAs were amplified using Amino Allyl MessageAmp™ II aRNA Amplification Kit (Ambion #AM1753, CA, USA) from 1 μg total RNA and directed labeled by NHS-CyDye (Cy5, Amershan) to uridine (aa-dUTP). The pre-hybridization of MOA v2 arrays were rehydrated by 100% ethanol following with deionized water. After the pre-hybridization, Cy5-labeled aaRNA was hybridize on MOA v2 in duplication (two arrays for each sample) at 50 °C for 16 hours in the presence of the Phalanx OneArray hybridization buffer. The arrays were scanned by Axon 4000B scanner (635 nm Power100, PMT 500; 532 nm Power10, PMT 460), and the fluoresence intensity was quantified. The data processing was carried out by Rosetta Resolver^®^ System (Rosetta Biosoftware, USA). We filtered the spots in which flag = −50 within all arrays and control probes. Probes passed filter were normalized by median scaling to remove systemic effect, then combine the duplicate data by averaging their intensity value. The Matrix value is normalized intensities. The normalized intensities and pairwised comparison were performed by Rosetta Resolver^®^ System error-weighted modeling to find significant diffrential expressed probes (log2 |Fold change| ≥ 1 and P < 0.05). Data have been deposited at the NCBI gene expression omnibus (GEO; http://www.ncbi.nlm.nih.gov/geo/) with GEO accession number GSE63615.

### Immunoblotting and antibodies

Briefly, samples of proteins from cytoplasm, nucleus, or whole cell were prepared as previous description^41^ and were resolved by SDS-PAGE and transferred to a PVDF membrane. The membrane was blocked for 1.5 hr in 5% non-fat milk and incubated with primary antibodies for Wnt1 (1:500; Abcam, Cambridge, UK), Wnt3a (1:1000; Abcam), β-catenin (1:200; Santa Cruz Biotechnology, CA, USA), phosphorylated β-catenin (Thr 41/Ser 45; 1:1000; Cell Signaling Technology, Beverly, MA), active β-catenin (Millipore, Temecula, CA) CD44 (1:1000; Abcam), Axin2 (1:1000; Abcam), GSK3β (1:200; Santa Cruz Biotechnology), phosphorylated GSK3β (Ser 9; 1:1000; Cell Signaling Technology), Akt (1:1000; Cell Signaling Technology), phosphorylated Akt (Ser 473; 1:2000; Cell Signaling Technology), FAK (1:500; Santa Cruz Biotechnology), phosphorylated FAK (Thr 397; 1:1000; Cell Signaling Technology), BMP2 (1:500; Abcam) or GAPDH (1:500; Santa Cruz Biotechnology). The membranes were then incubated with horseradish peroxidase-conjugated anti-mouse or anti-rabbit IgG (1:10,000; Zhongshan Golden Bridge Biotechnology Co., Beijing, China). Finally, the membranes were developed using a Kodak medical X-ray processor (Kodak, Rochester, NY, USA).

### Real-time PCR

Cells were collected and total mRNA was isolated using Trizol (Invitrogen, Carlsbad, CA, USA). Real-time PCR quantitative mRNA analyses were performed with a Mastercyler^®^ ep realplex (Eppendorf, Germany) using SYBR^®^ Green Realtime PCR Master Mix (Toyobo, Osaka, Japan). The primers are for mouse *wnt1* (sense: 5′-TCGAGAAATCGCCCAACTTC-3′; anti-sense 5′-TGCAAGCTCGTCCAGCTGT-3′), mouse GAPDH (sense: 5′-TGCACCACCAACTGCTTA- G-3′; anti-sense 5′-GGATGCAGGGATGATGTT- C-3′).

### Immunocytochemical Staining

Cells were cultured for 4 d on the stiff or soft gel-coated slides, and fixed with 4% paraformal- dehyde. After blocking in blocking buffer (containing 3% goat serum, 10% horse serum, 0.3% Triton X-100 in PBS) for 30 min at room temperature, the slides were incubated with primary antibodies against Sox9 (1:100; Abcam), Aggrecan (1:50; Abcam), ColII (1:50; Abcam), Runx2 (1:100), ColI (1;100), MAP2 (1:500; Abcam) , Nestin (1:100; Abcam) or NFL (1:500; Abcam), then with anti-mouse IgG conjugated with FITC (1:4,000; Abcam), anti-rabbit IgG conjugated with TRITC (1:4,000; Abcam) or anti-goat IgG conjugated with TRITC (1:4,000; Abcam). Finally, cell nuclei were visualized with DAPI (Sigma-Aldrich, St. Louis, Missouri, USA) and viewed under a Leica TCS SP5 confocal microscopy system (Leica, Germany).

### Chromatin immunoprecipitation

The assay was done according to instruction of Upstate ChIP assay kit (Upstate, NY, USA). Briefly, primary chondrocytes were treated for 24 hr with 20 mM LiCl or NaCl and then cross-linked with 1% formaldehyde at 37 °C for 10 min. After centrifugation at 4 °C for 4 min at 2000 rpm, the cells were suspended with lysis buffer and sonicated three times. The supernatant was collected and diluted with ChIP dilution buffer, followed by immunoclearing with salmon sperm DNA/protein A agarose at 4 °C for 1 hour. Then, anti-β-catenin antibody (Santa Cruz Biotechnology) was added to immunoprecipitate the chromatin overnight. The immunoprecipitation complex was eluted and heated at 65 °C for 4 hours. After digestion with proteinase K, DNA was obtained by phenol/chloroform/isoamyl alcohol extraction and used as PCR template.

### Plasmid constructs and transfection

Negative control pGL3-Basic was purchased from Promega (WI, USA). To construct the plasmid pWNT, a 679-bp fragment containing the putative binding site of β-catenin in WNT promoter (bp −615 to +63, numbered according to the transcription start site where bp +1 is A in ATG) was generated by PCR amplification with the primer pair: forward 5′-GCACTCGAGCAGAGGAGACGGACTTCGAG-3′, and reverse 5′-TACAAGCTTGAGCGGTCAGTGCCAGTAGC-3′, and cloned into the pGL3-Basic. The plasmid pWNTm that contains mutagenesis of the putative binding site in WNT promoter was generated by site-directed mutagenesis PCR using the plasmid pWNT as a substrate. The 6 bp of WNT promoter (bp −352 to −347) was deleted with the following mutagenic primers: 5′-G-GTTAGCCTGTCAGCTTCAGACCGGCAAGA-3′, 5′-TCTTGCCGGTCTGAAGCTGACAGG-CTAACC-3′. Transfection of the reporter constructs into MC-3T3 cells was performed with Lipofectamin 2000 (Invitrogen, CA. USA.) according to the manufacturer’s instructions.

### siRNA knockdown

At 24 hr before transfection, chondrocytes were plated onto six-well culture plates and transfected with siRNAs at 70–80% confluence. siRNAs for β-catenin was purchased from Santa Cruz Biotechnology (sc-29210). siRNAs for Intigrin β1, Intigrin α1, Intigrin α10, FAK, ILK, YAP/TAZ and control scrambled siRNA were purchased from GenePharma. Chondrocytes were washed in PBS and resuspended in resuspension buffer R included with Neon™ kit (Invitrogen). Transfections of siRNA into chondrocytes with Neon Transfection System (Invitrogen) were performed according to the manufacturer’s protocol with two pulses of 1400 V and 20 ms. Transfection efficiency was assessed using qPCR and western blot (Data not shown). Eletroporated cells were resuspended in culture medium containing serum and supplements without antibiotics and incubated for 24 hr at 37 °C in a humidified 5% CO_2_ incubator.

### Luciferase assay

MC-3T3 cells cultured in 24-well plates were transiently transfected with a mixture containing 1 μg of reporter plasmid and 5 ng of pRL-CMV. After 6 hous of transfection, the medium was changed and the cells were treated with 20 mM LiCl or NaCl for 18 hours. Firefly and Renilla luciferase activities were measured using the Dual Luciferase Kit (Promega, WI, USA). The firefly luciferase values of each sample were normalized by Renilla luciferase activity and data were reported as relative light units.

### Proteins and chemicals

Wnt1, Wnt3a and WIF were purchased from Abcam (Cambridge, UK). sFRP1 was purchased from Sino Biological Inc. BIO, Akti-1/2, Cardamonin, NaCl, LiCl and DMSO were purchased from Sigma-Aldrich (St. Louis, MO, USA). PMA was purchased from Santa Cruz Biotechnology (CA, USA). PF573228 was purchased from Tocris Bioscience (Bristol, UK). Anti-integrin β1 (clone HM β1-1) and control IgG (clone HK888) were purchased from Biolegend (San Diego, CA, USA).

### Statistical Analysis

Data are presented as mean ± SEM. Student’s *t*-test was used to compare differences between two experimental groups. ANOVA analysis was used when the comparison was made using any group more than once. When a significant difference was found by ANOVA, the Student– Neumann–Keuls method was used post hoc to determine the significance of differences between groups.

## Additional Information

**How to cite this article**: Du, J. *et al.* Extracellular matrix stiffness dictates Wnt expression through integrin pathway. *Sci. Rep.* **6**, 20395; doi: 10.1038/srep20395 (2016).

## Supplementary Material

Supplementary Information
