# Supplementary material for: Extracellular matrix stiffness dictates Wnt expression through integrin pathway
Source: Sci Rep. 2016 Feb 8;6:20395. doi: 10.1038/srep20395 (PMC4745056; doi:10.1038/srep20395)
Supplement: Supplementary Information [file srep20395-s1.doc]

**Supporting Information**

**Extracellular matrix stiffness dictates Wnt expression through integrin pathway***

**Jing Du, Yan Zu,** **Jing Li,** **Shuyuan Du,** **Yipu Xu,** **Lang Zhang,** **Li Jiang,Zhao Wang, Shu Chien and Chun Yang**

**Supplementary table 1. Oligonucleotide array profiles for genes in chondrocytes significantly regulated by ECM stiffness.**

| **Gene Description** | **Symbol** | **Stiff/Soft** | **p-value** |
| --- | --- | --- | --- |
| Cytoskeleton |  |  |  |
| FERM and PDZ domain containing 1 | Frmpd1 | 0.496922 | 0.00012 |
| LIM domain binding 3 | Ldb3 | 0.767835 | 0.011721 |
| cysteine and glycine-rich protein 3 | Csrp3 | 0.423393 | 5.81E-24 |
| formin 1 | Fmn1 | 1.53546 | 0.000291 |
| keratin 19 | Krt19 | 2.098956 | 4.16E-08 |
| keratin 6A | Krt6a | 1.719939 | 5.98E-05 |
| keratin associated protein 13 | Krtap13 | 1.576584 | 0.018596 |
| myomesin 1 | Myom1 | 0.457699 | 7.73E-06 |
| myosin light chain, phosphorylatable, fast skeletal | Mylpf | 0.477417 | 0.011051 |
| myosin, heavy polypeptide 11, smooth muscle | Myh11 | 1.630952 | 0.048694 |
| myozenin 2 | Myoz2 | 0.469226 | 2.31E-07 |
| pericentriolar material 1 | Pcm1 | 1.798658 | 0.049017 |
| protein tyrosine phosphatase, non-receptor type 4 | Ptpn4 | 1.684486 | 0.038789 |
| rabphilin 3A | Rph3a | 1.573403 | 0.014712 |
| tektin 5 | Tekt5 | 0.443145 | 6.93E-07 |
| Membrane |  |  |  |
| C-type lectin domain family 7 | Clec7a | 1.653044 | 0.004298 |
| DnaJ (Hsp40) homolog, subfamily C, member 14 | Dnajc14 | 1.554575 | 0.000098 |
| G protein-coupled receptor 126 | Gpr126 | 1.631462 | 0.000002 |
| G protein-coupled receptor 64 | Gpr64 | 1.678787 | 0.000003 |
| GRIP and coiled-coil domain containing 2 | Gcc2 | 1.57739 | 0.048698 |
| RIKEN cDNA 1110032E23 gene | Fam198b | 1.58513 | 0.033005 |
| S100 calcium binding protein A9 (calgranulin B) | S100a9 | 0.432937 | 0.00047 |
| SLIT and NTRK-like family, member 2 | Slitrk2 | 1.531295 | 0.014279 |
| TM2 domain containing 3 | Tm2d3 | 0.712801 | 0.000955 |
| acyl-Coenzyme A binding domain containing 5 | Acbd5 | 1.606521 | 0.004873 |
| adaptor protein complex AP-1, mu 2 subunit | Ap1m2 | 1.66431 | 0.012013 |
| aspartate-beta-hydroxylase | Asph | 1.265435 | 0.034405 |
| calcium channel, voltage-dependent, L type, alpha 1S subunit | Cacna1s | 0.450475 | 0.000001 |
| carbonic anhydrase 14 | Car14 | 0.435701 | 0.000002 |
| cerebellin 1 precursor protein; similar to recerebellin-1 | Cbln1 | 0.335577 | 3.00E-08 |
| chondrolectin | Chodl | 0.27984 | 3.13E-07 |
| claudin 11 | Cldn11 | 0.415533 | 0.000001 |
| cytochrome c oxidase, subunit VIIa 2 | Cox7a2 | 1.652581 | 0.000157 |
| early endosome antigen 1 | Eea1 | 1.633967 | 0.019229 |
| expressed sequence AI480653 | AI480653 | 1.534032 | 0.000015 |
| fatty acyl CoA reductase 1 | Far1 | 1.64112 | 0.001739 |
| fatty acyl CoA reductase 2 | Far2 | 1.805165 | 0.000097 |
| fibronectin type III domain containing 5 | Fndc5 | 0.494935 | 0.000055 |
| filamin A interacting protein 1-like | Filip1l | 1.535376 | 0.01819 |
| gamma-aminobutyric acid (GABA) A receptor, subunit alpha 1 | Gabra1 | 1.547209 | 0.032037 |
| gap junction membrane channel protein epsilon 1 | Gje1 | 2.190195 | 0.039584 |
| glycoprotein 49 A | Lilrb4 | 1.612469 | 0.002184 |
| golgi integral membrane protein 4 | Golim4 | 1.5222 | 0.007117 |
| guanylate binding protein 5 | Gbp5 | 1.85621 | 0.03943 |
| heparan sulfate (glucosamine) 3-O-sulfotransferase 5 | Hs3st5 | 1.560418 | 0.002591 |
| heparan sulfate 6-O-sulfotransferase 2 | Hs6st2 | 1.83641 | 0.001088 |
| integrin alpha X | Itgax | 1.825624 | 0.000028 |
| interleukin 18 receptor 1 | Il18r1 | 1.502756 | 0.001803 |
| kinectin 1 | Ktn1 | 1.662125 | 0.014929 |
| lin-7 homolog C (C. elegans) | Lin7c | 1.532763 | 0.000344 |
| lymphocyte antigen 9 | Ly9 | 1.533106 | 0.011452 |
| lymphotoxin B receptor | Ltbr | 1.804529 | 0.000016 |
| membrane-spanning 4-domains, subfamily A, member 8A | Ms4a8a | 1.503524 | 0.006832 |
| met proto-oncogene | Met | 1.506159 | 0.000311 |
| monoamine oxidase B | Maob | 1.798233 | 0.0034 |
| neurexin III | Nrxn3 | 2.052355 | 0.037409 |
| olfactory receptor 867 | Olfr867 | 1.502851 | 0.023163 |
| platelet endothelial aggregation receptor 1 | Pear1 | 1.684649 | 0.001387 |
| pre T-cell antigen receptor alpha | Ptcra | 1.506098 | 0.034927 |
| prostaglandin-endoperoxide synthase 2 | Ptgs2 | 1.669964 | 0.009752 |
| protocadherin 17 | Pcdh17 | 1.548315 | 0.005364 |
| protocadherin 19 | Pcdh19 | 1.620628 | 0.000005 |
| protocadherin 7 | Pcdh7 | 1.524159 | 0.000104 |
| radixin | Rdx | 1.622028 | 0.000239 |
| regulator of G-protein signaling 17 | Rgs17 | 1.62213 | 0.046371 |
| ring finger and CCCH-type zinc finger domains 2 | Rc3h2 | 1.648416 | 0.00139 |
| scavenger receptor class A, member 5 (putative) | Scara5 | 0.498657 | 0.004005 |
| selection and upkeep of intraepithelial T cells 5 | LOC639826 | 1.631163 | 0.013897 |
| serum deprivation response | Sdpr | 1.625068 | 0.000015 |
| similar to odorant response abnormal 4 | BC003331 | 1.549142 | 0.044344 |
| solute carrier family 4 (anion exchanger), member 4 | Slc4a4 | 1.696702 | 0.000026 |
| solute carrier organic anion transporter family, member 2a1 | Slco2a1 | 1.518038 | 0.0003 |
| sushi domain containing 3 | Susd3 | 0.475328 | 5.48E-08 |
| thioredoxin-related transmembrane protein 3 | Tmx3 | 1.56345 | 0.004427 |
| thyrotropin releasing hormone receptor | Trhr | 1.699922 | 0.002689 |
| transient receptor potential cation channel, subfamily M, member 7 | Trpm7 | 1.749005 | 0.000965 |
| transmembrane 4 superfamily member 1 | Tm4sf1 | 1.774456 | 0.000357 |
| transmembrane 7 superfamily member 4 | Tm7sf4 | 1.818093 | 0.002128 |
| transmembrane emp24 protein transport domain containing 5 | Tmed5 | 2.165914 | 0.000211 |
| transmembrane protein 106B | Tmem106b | 1.531776 | 0.023119 |
| transmembrane protein 188 | Tmem188 | 1.840078 | 0.000189 |
| tripartite motif-containing 72 | Trim72 | 0.486949 | 1.22E-12 |
| unc-84 homolog A (C. elegans) | Sun1 | 1.561833 | 0.00035 |
| vacuolar protein sorting 37A (yeast) | Vps37a | 1.942348 | 0.033596 |
| Cell cycle |  |  |  |
| CD2-associated protein | Cd2ap | 1.882862 | 0.000082 |
| RIKEN cDNA 4632434I11 gene | 4632434I11Rik | 1.629941 | 0.029287 |
| breast cancer 1 | Brca1 | 1.72256 | 0.012006 |
| caspase 8 associated protein 2 | Casp8ap2 | 1.580857 | 0.027114 |
| centromere protein E | Cenpe | 1.544016 | 0.044201 |
| integrin beta 1 (fibronectin receptor beta) | Itgb1 | 1.551419 | 0.000003 |
| leucine rich repeat and coiled-coil domain containing 1 | Lrrcc1 | 1.629061 | 0.041862 |
| peptidylprolyl cis/trans isomerase, NIMA-interacting 1-like | Pin1 | 1.591867 | 0.000706 |
| predicted gene 9481; mitochondrial ribosomal protein L41 | Mrpl41 | 0.768292 | 0.026609 |
| spindle assembly 6 homolog (C. elegans) | Sass6 | 1.624037 | 0.005389 |
| stromal antigen 2 | Stag2 | 1.529916 | 0.003868 |
| structural maintenance of chromosomes 4 | Smc4 | 1.814491 | 0.027247 |
| Wnt pathway |  |  |  |
| wingless-related MMTV integration site 1 | wnt1 | 1.223844 | 0.033522 |
| wingless-related MMTV integration site 2b | wnt2b | 1.572785 | 0.022918 |
| wingless-related MMTV integration site 3a | wnt3a | 1.278601 | 0.019586 |
| wingless-related MMTV integration site 6 | wnt6 | 1.305089 | 0.02075 |
| Wnt inhibitory factor 1 | wif1 | 0.540211 | 0.030506 |
| vascular endothelial growth factor A | vegfa | 1.402611 | 0.010346 |
| vascular endothelial growth factor B | vegfb | 8.364928 | 0.025055 |
| CD44 antigen | cd44 | 1.291274 | 0.025679 |
| axin2 | axin2 | 1.447694 | 0.006689 |

**Supplementary figure 1.**


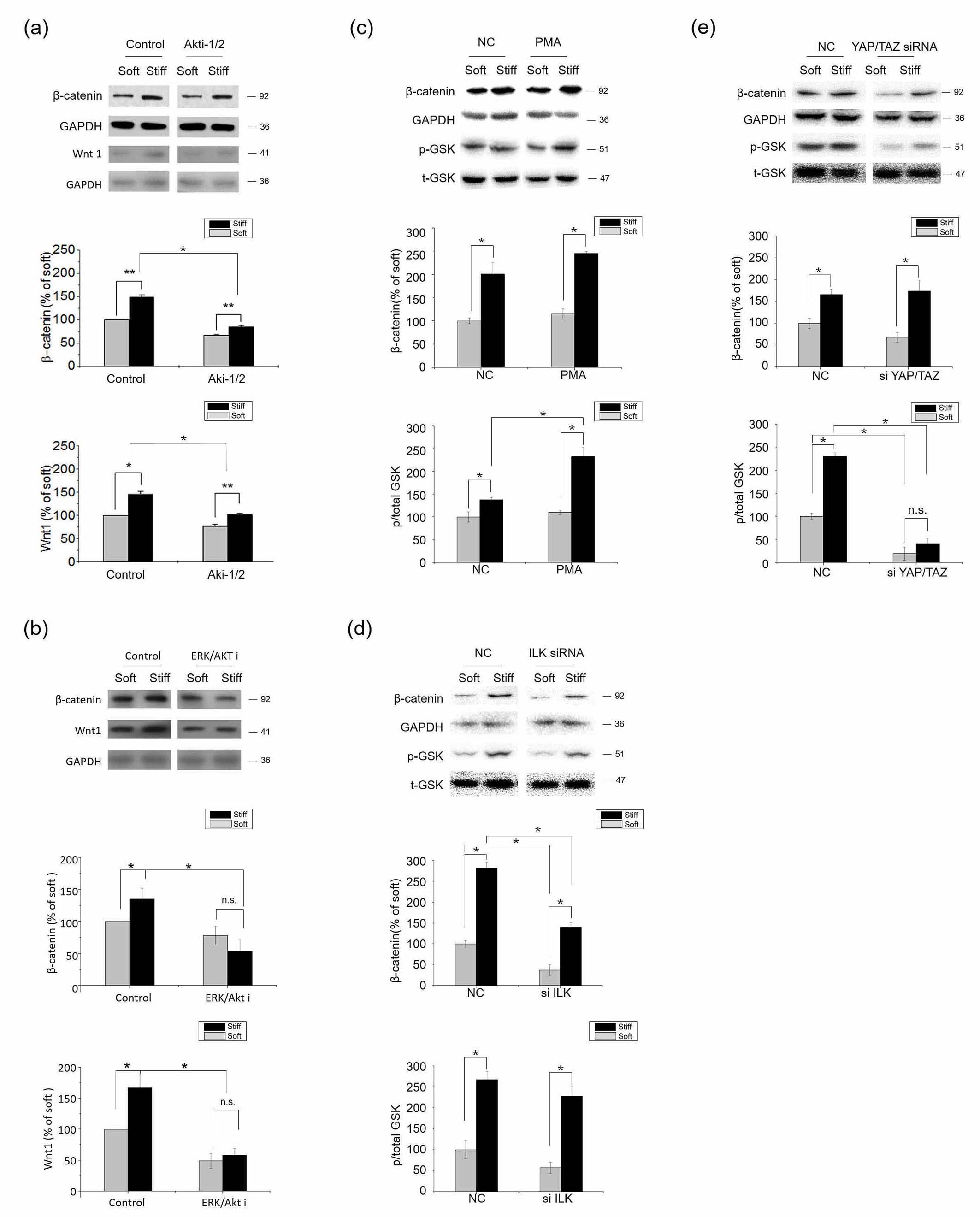


**Supplementary figure 1. The downstream regulatory pathways of integrin.** (a) Total β-catenin and Wnt1 levels in the presence of Akti-1/2 (10 μM) or DMSO were analyzed by western blotting. (b) Total β-catenin and Wnt1 levels in chondrocytes 48 hr after seeding on the stiff or the soft ECM in the presence of both Erk inhibitor PD98059 (50μM) and Akti-1/2 (10 μM) or DMSO were analyzed by western blotting. (c) Total β-catenin and phosphorylated GSK3β levels in the presence of PMA (100 nM) or DMSO were analyzed by western blotting. (d) β-catenin and phosphorylated GSK3β levels in chondrocytes48 hr after seeding on the stiff or the soft ECM in the presence of ILK siRNA or scramble were analyzed by western blotting. (e) β-catenin and phosphorylated GSK3β levels in chondrocytes48 hr after seeding on the stiff or the soft ECM in the presence of YAP/TAZ siRNA or scramble were analyzed by western blotting. Western results were from 3 independent experiments, with blots exemplifying one experiment and the bar graphs showing the combined results on stiff matrix expressed as percentages (means ± SEM) of the corresponding results on soft matrix. GAPDH was used to normalize for equal loading in western blotting. * P < 0.05, ** P < 0.01, n.s. stands for not statistically significant.

**Supplementary figure 2.**


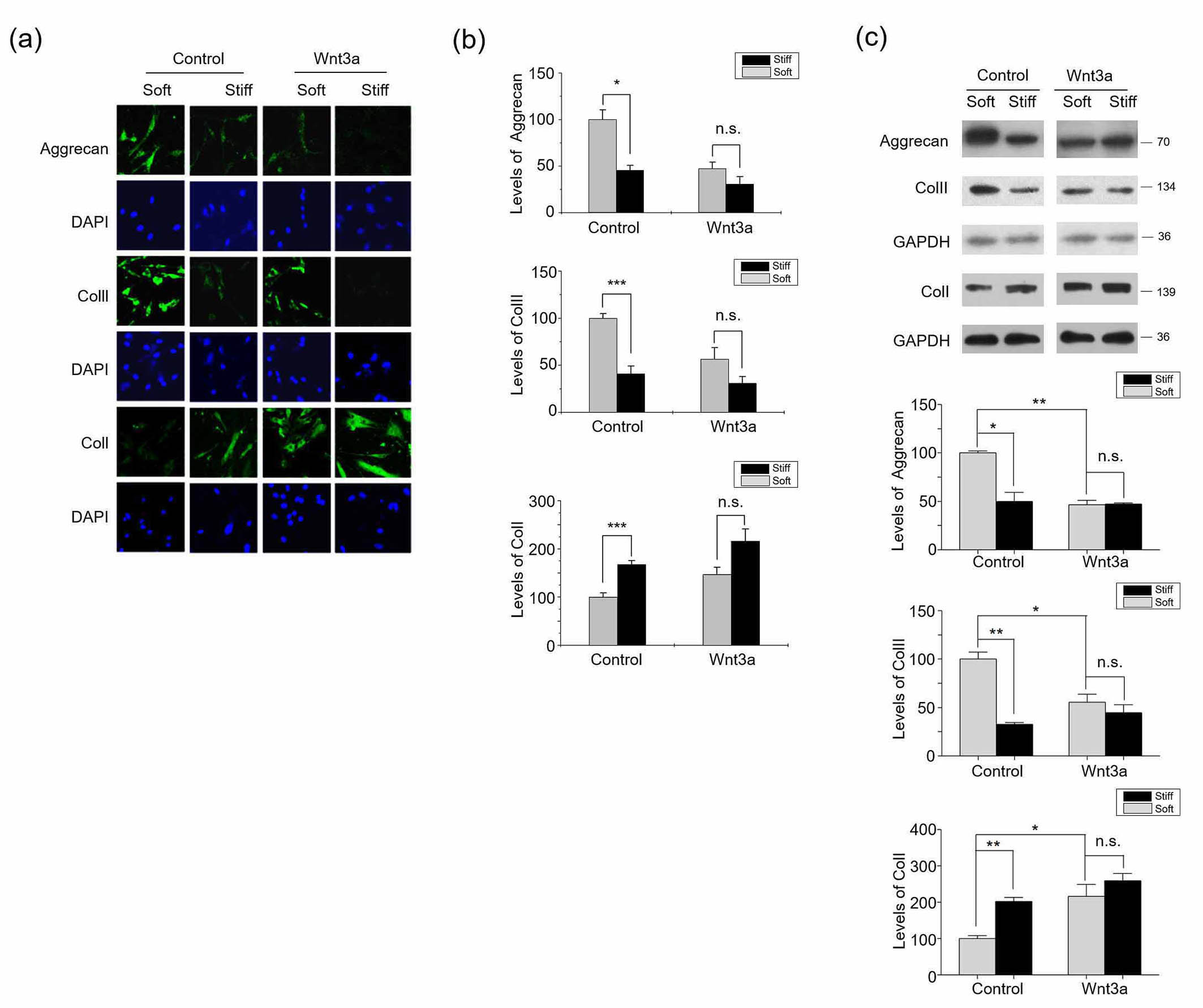


**Supplementary figure 2. Wnt3a contributes to ECM stiffness regulating chondrocytes phenotype maintenance.** (a) Chondrocytes were cultured on the stiff or the soft ECM for seven days in the presence of Wnt3a (100 ng/ml) or solvent, followed by determination of Aggrecan, ColII and ColI expressions by immunocytochemical staining. Scale Bar: 30 μm. (b) is the statistic result of (a). (c) Chondrocytes were cultured on the stiff or the soft ECM for seven days in the presence of Wnt3a (100 ng/ml) or solvent, followed by determination of Aggrecan, ColII and ColI expressions by western blotting. Western results were from 3 independent experiments, with blots exemplifying one experiment and the bar graphs showing the combined results on stiff matrix expressed as percentages (means ± SEM) of the corresponding results on soft matrix. GAPDH was used to normalize for equal loading in western blotting. * P < 0.05, ** P < 0.01, n.s. stands for not statistically significant.
